# MicroRNA-130a inhibits growth and metastasis of osteosarcoma cells by directly targeting ZEB1

**DOI:** 10.3892/mmr.2022.12756

**Published:** 2022-05-31

**Authors:** Lankai Yi, Meixiu Liu, Zhiliang Tang

Mol Med Rep 16: 3606–3612, 2017; DOI: 10.3892/mmr.2017.6968

Following the publication of this paper, it was drawn to the Editors’ attention by a concerned reader that various panels showing cell migration assay data in Figs. 3B and C and 5C and D were strikingly similar to data appearing in different form in other articles by different authors. Furthermore, overlapping data panels were identified within this paper comparing the cell migration assay images between Figs. 3B and 5C. Owing to the fact that the contentious data in the above article had already been published elsewhere, or were already under consideration for publication, prior to its submission to *Molecular Medicine Reports*, the Editor has decided that this paper should be retracted from the Journal. The authors were asked for an explanation to account for these concerns, but the Editorial Office did not receive a reply. The Editor apologizes to the readership for any inconvenience caused.

